# Bonding Effect of Partial Valence Band Depletion in Metallic [ZnSi] Layers of Li_2_[ZnSi] and a Comparison to “White Graphene” h‑BN

**DOI:** 10.1021/acs.inorgchem.5c03292

**Published:** 2025-09-30

**Authors:** Frank R. Wagner, Alim Ormeci, Ulrich Schwarz

**Affiliations:** Chemical Metals Science, Max Planck Institute for Chemical Physics of Solids, Nöthnitzer Str. 40, Dresden 01187, Germany

## Abstract

The compound Li_2_[ZnSi] is known to crystallize in the Li_2_CuAs structure type featuring graphite-like [ZnSi] layers separated by Li atoms, thus supporting the view of a Zintl phase (Li^+^)_2_[ZnSi]^2–^. The comparative band structure (PBE + *U* technique) and chemical bonding analysis with insulating h-BN using the electron localizability indicator ELI-D and the electron density reveal unexpected crystal structure–band structure effects. They lead to partial valence band depletion and the metallic conductivity of Li_2_[ZnSi]. As a result, the conventional polar diatomic bonds of h-BN become polar 4-atomic ones in Li_2_[ZnSi].

## Graphical abstract

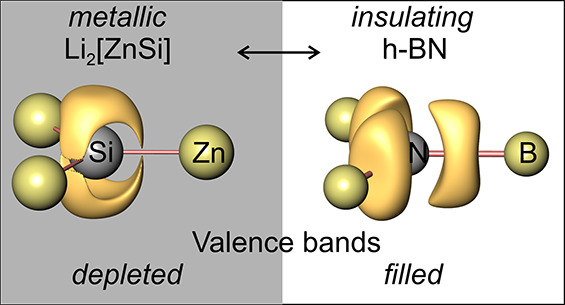

## Introduction

The outstanding physical properties of graphene have stimulated widespread scientific interest in compounds featuring graphene-related layers in general. Their exceptional relevance is reflected in the use of the label “white graphene” for the related binary variety hexagonal boron nitride h-BN. Beyond that, the occurrence of heteroatomic networks holding the same pattern of condensed six-membered rings is observed in polar intermetallic compounds like Li_2_[CuAs],^1^ β-Li_2_[ZnGe],^2^ Li_2_[CuP],^3^ and Li_2_[ZnSi].^4^

In Li_2_[ZnSi], the two-dimensional network is formed by silicon and zinc atoms. In this context, it is important to note that in thermodynamic equilibrium, Zn and Si do not form any binary intermetallic phase in the solid. Thus, the Li atoms may be considered to take the role of a mediator between those atomic constituents, which has been utilized to synthesize a metastable amorphous Zn–Si binary phase.^5^ The Li-mediated atomic arrangement in Li_2_[ZnSi] represents a heterographite variety [ZnSi] in which the layers are separated by lithium atoms. Alternatively, the crystal structure of Li_2_ZnSi has been also described as a coloring variant of Na_3_As,^6^ where the Si atoms replace As.

With regard to chemical bonding, any description along the lines of the Zintl concept assumes the transfer of the valence electrons of lithium to the zinc–silicon partial structure. However, the chemical bonding and charge distribution within the [ZnSi]^2–^ nets defy easy categorization, especially as they involve the transition metal zinc. Two qualitative options may be discussed as limiting cases. A complete transfer of the valence electrons of Li to Zn atoms would result in Zn^2–^ anions and Si^0^ atoms holding four valence electrons each such that each layer atom was isoelectronic to carbon in graphene. Considering the small electronegativity difference between Zn and Si, a more realistic scenario would be the equal distribution of the transferred charge according to (Li^+^)_2_[Zn^–^Si^–^] bringing about an electron distribution that is formally analogous to that in boron nitride B^0^N^0^. The experimentally observed metallic properties of Li_2_[ZnSi][Bibr ref4] correspond to those computed for isotypic Li_2_CuP,^7^ while isoelectronic Na_2_MgSn is characterized as a narrow gap semiconductor.^8^ The hexagonal modification of boron nitride h-BN is an insulator (”white graphene”^9^) with a band gap of approximately 6 eV.^9^

In order to investigate the relationship between the ternary intermetallic compound and binary h-BN, the present study compares the electronic structures and the chemical bonding scenarios in both compounds by means of band structure analysis and within the framework of the combined electron localizability-QTAIM (Quantum Theory of Atoms in Molecules^10^) approach in real space.^11^

## Computational Section

The all-electron full-potential local orbital (FPLO)^12^ method was employed to perform first-principles electronic structure calculations on Li_2_[ZnSi] and h-BN. The generalized gradient approximation (GGA) to the density functional theory as parametrized by Perdew, Burke, and Ernzerhof (PBE) was used.^13^ The effect of strong electron correlations within Zn­(3d^10^) states was investigated by employing the PBE + *U* method (fully localized limit^14^) with *U* values of 0, 4, 6, 10, and 12 eV.

The idealized structure model of Li_2_[ZnSi] without stacking faults^4^ was employed (Table 1) with a 22 × 22 × 12 *k*-mesh. For h-BN, the literature data^15^ of the crystal structure were used (Table 1). Investigation of chemical bonding in position space was carried out by combining topological analyses of the electron localizability indicator (ELI-D) and the electron density (ED) distributions.^11^ The topological analysis of the ED distribution is based on Bader’s quantum theory of atoms in molecules (QTAIM).^10^ The ED and the σ-spin ELI-D distributions, ρ­(**r**) and Υ_D_^σ^(**r**), respectively, were calculated on a uniformly spaced three-dimensional grid in position space with 1.67 pm mesh size by a module implemented in the FPLO package.^16^ The negative relative ELI-D Laplacian distribution −∇^2^Υ_D_^σ^(**r**)/Υ_D_^σ^(**r**) was calculated from the ELI-D one by the differences method. The topological analyses were done using the program DGrid.^17^ The ELI-D/QTAIM basin intersection method was utilized to determine the atoms contributing to the electronic populations of the ELI-D basins.^11^

The shapes of the QTAIM atomic basins were employed to compute topological coordination numbers (*tCN*s)^18^ for all sub-coordination scenarios identified. For this purpose, the solid angles subtended by the triangulated diatomic contact surfaces^19^ at the nuclear positions were evaluated using program system QTopCN.^20^

## Results and Discussion

### Structure Model

The Zn and Si atoms of the ordered Li_2_[ZnSi] structure model^4^ used herein (crystallographic parameters in Table 1) are alternatingly arranged in planar hexagonal layers perpendicular to the *c*-axis, which are separated from each other by Li atoms (Figure 1a). Like in graphite, the _∞_^2^[ZnSi] layers display a stacking sequence *AB*. The Li atoms are arranged above and below the center of each Zn_3_Si_3_ hexagon and form puckered 6^3^ nets. The _∞_^2^[Li_2_] nets are arranged along the [001] as layers of types *C* and *C’* (Figure 1a). The stacking sequence of 6^3^ layers would then be denoted as *ACBC’*. This corresponds to the *ABCB* stacking sequence in the 4H-SiC polytype, where close packed 3^6^ nets are stacked instead of their dual 6^3^ nets. Summarizing the characteristics of the Li_2_[ZnSi] structure, all atoms are situated within two different columns along [001]: Li and Si atoms are located along column (1/3, 2/3, *z*), while the Zn atoms occupy positions along column (0, 0, *z*) without the first nearest neighbor (1nn) Li contacts in this direction, thus opening a path for two second nearest neighbor (2nn) Zn–Zn^*z*^ contacts (Table 2).

**Table 1:** Crystallographic Parameters for the Li_2_[ZnSi][Bibr ref4] and h-BN^15^ structures employed^a^

species	Wyckoff site	site symm.	position
Li	(4*f*)	3*m*.	1/3, 2/3, 0.917563
Zn	(2*b*)	6̅*m*2	0, 0, 1/4
Si	(2*c*)	6̅*m*2	1/3, 2/3, 1/4
B	(2*c*)	6̅*m*2	1/3, 2/3, 1/4
N	(2*d*)	6̅*m*2	1/3, 2/3, 3/4

a Space group *P*6_3_/*mmc* (#194); lattice parameters Li_2_[ZnSi]: *a* = 424.6 pm, *c* = 822.85 pm; BN: *a* = 250.4323 pm, *c* = 665.8852 pm.

**Table 2:** Selected Interatomic Distances in Li_2_[ZnSi]^a^

contact (1nn)	distance (pm)	contact (2nn)	distance (pm)
*d*(Si–Zn)*^xy^*	245 (3x)	*d*(Zn–Zn)*^z^*	411 (2x)
*d*(Si–Li)^z^	274 (2x)	*d*(Zn–Zn)*^xy^*	425 (6x)
*d*(Si–Li)*^xyz^*	281 (6x)	*d*(Si–Si)^xy^	425 (6x)
*d*(Zn–Li)*^xyz^*	281 (6x)	*d*(Si–Si)*^xyz^*	479 (6x)
		*d*(Si–Zn)*^xyz^*	479 (6x)

a Indicated frequencies of contact types (in parentheses) of distances *d*(*A*–*B*) are enumerated for species *A*. Superscripts *x*, *y*, and *z* indicate the nonzero components of the distance vectors of all neighbors of the type denoted.

**Figure 1:**
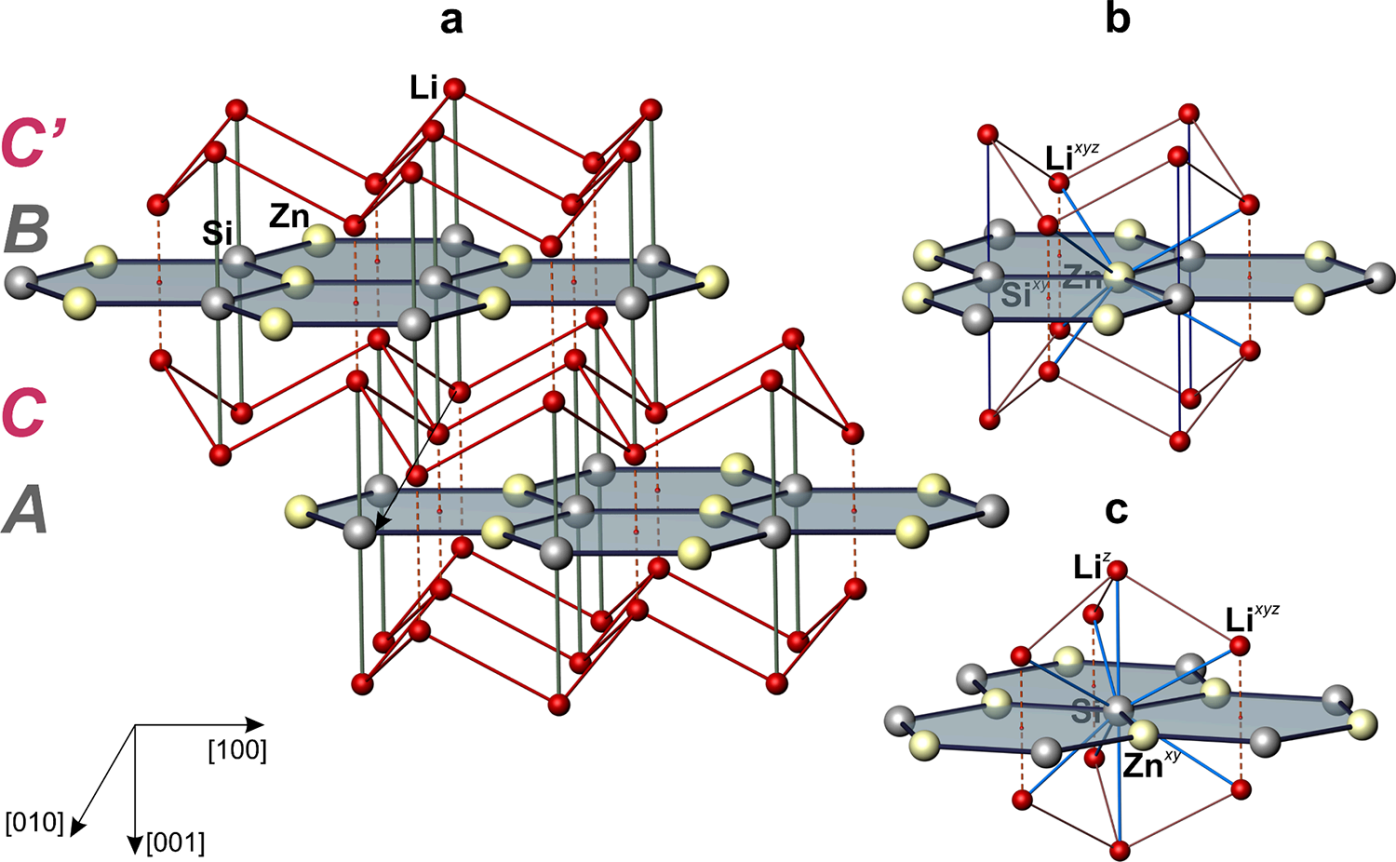
Crystal structure of Li_2_[ZnSi]. (a) *AB-*type stacking sequence of planar _∞_^2^[ZnSi] 6^3^ nets being separated by puckered _∞_^2^[Li_2_] 6^3^ nets occupying layer types *C* and *C’*; (b, c) first nearest neighbor (1nn) environment (thick blue lines) of Zn and Si; related interatomic distances are given in Table 2.

Tentative atomic environments of species Zn and Si are displayed in Figure 1b,c, respectively, and associated interatomic distances are given in Table 2. A comprehensive study of coordination environments on the basis of the calculated electron density distribution is reported below.

### Electronic Structure and Chemical Bonding

#### Electronic DOS

The total electronic density of states (DOS), the atom-projected DOS, and the electron bands along high-symmetry lines are computed for the structure (Figure 1) specified in Table 1. The total DOS (Figure 2 top) shows the compound to be a metal, in agreement with the experimentally observed temperature dependence of electrical conductivity.^4^ The electronic states at low energies between −9.6 and −8.2 eV result from two nominal Si­(3s) bands (2 fu/unit cell) displaying strong admixtures of Zn­(3d) states. Around −7.5 eV, a steep DOS peak with a very narrow bandwidth of 0.25 eV is found. It contains 8 rather localized bands of mainly Zn­(3d) character. Although there is some small Si­(3p) mixing, these bands mostly reflect the atomic-like nature of the Zn­(3d) states. In the energy range from −7.32 to −6.5 eV, a DOS region is identified containing two further nominal Zn­(3d) bands such that, in sum, all nominal Zn­(3d^10^) states are occupied. However, these dispersive bands are formed mainly by mixing the Zn­(3d) and Si­(3s) states. They display a bandwidth of about 0.8 eV and represent Zn dominated counterparts of the nominal Si­(3s) bands. It is to be noted that this DOS region clearly reflects an initially unexpected participation of the Zn­(3d) states in chemical bonding, which is to be put into question and will be investigated below. The top region of the valence bands contains 6 bands and is dominated by Si­(3p) states. Zn contributions are found to increasingly mix in with rising energy, where Zn­(4s*)* contributions are greater than those of Zn­(4p*)* at the bottom of this region and smaller in the upper part. While DOS analysis confirms that Li_2_[ZnSi] is metallic, the nature of the gap and the mechanism responsible for the gap closing are encoded in the band structure, which is investigated in the following part.

**Figure 2:**
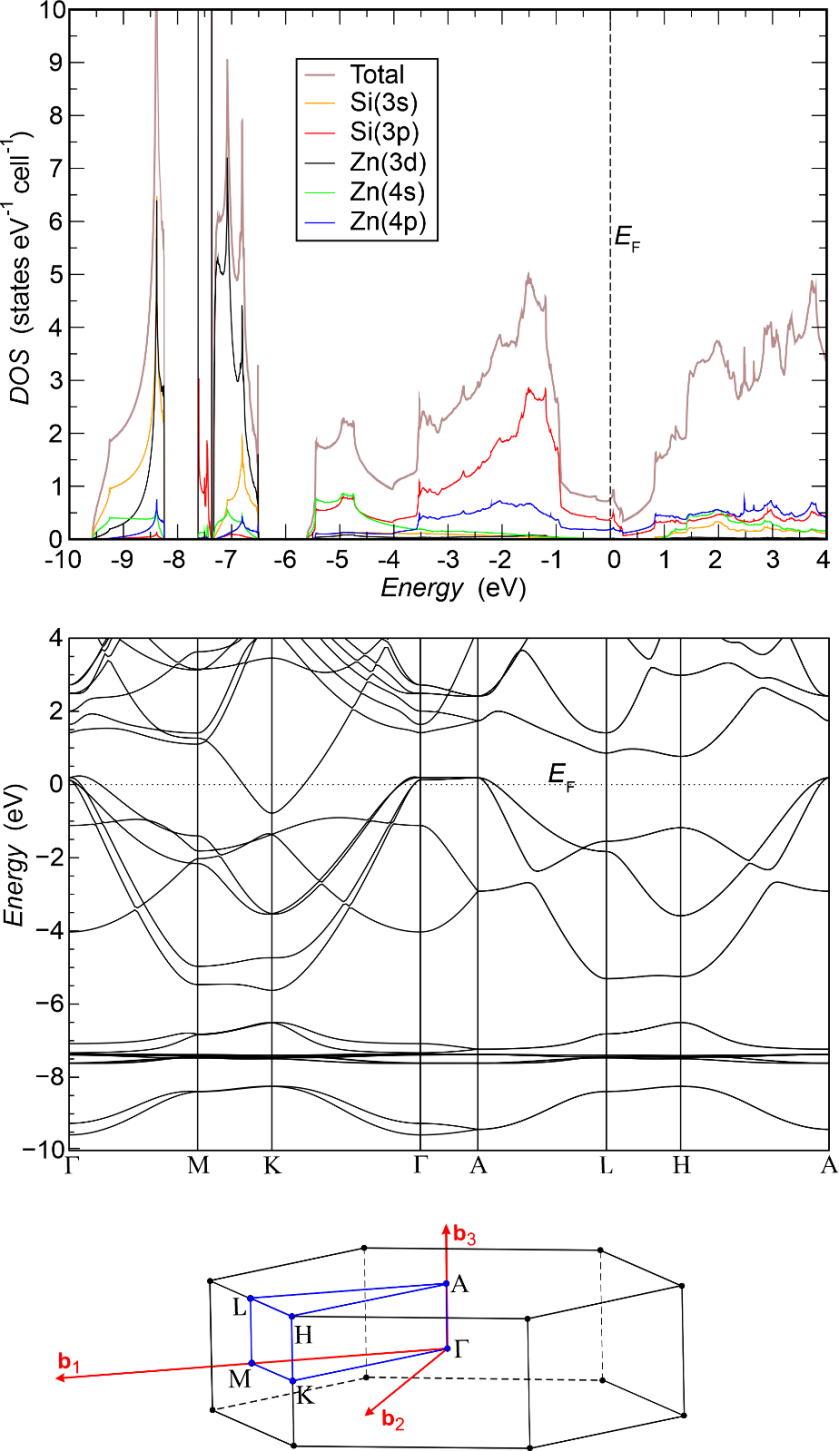
(top) Total and orbital-projected DOS for relevant Zn and Si states obtained for *U* = 0; (middle) electronic energy band dispersions (*U* = 0) along the high symmetry lines of the Brillouin zone; (bottom) first Brillouin zone of the hexagonal lattice.

#### Band Structure and Gap Scenario

The band structure of Li_2_[ZnSi] (Figure 2 middle) displays a valence band maximum (VBmax) along the **k** point line Γ-A and a conduction band minimum (CBmin) at K that is lower in energy than the VBmax. This negative indirect band gap yields the experimentally verified metallic conductivity of Li_2_[ZnSi].^4^ With the detailed band structure of the wide-gap insulator h-BN at hand (Figure 3 left), it is interesting to analyze how the negative indirect gap is created in Li_2_[ZnSi] and to compare it to the situation in h-BN, where a calculated band gap of about 4 eV is seen in the band structure (Figure 3).

**Figure 3:**
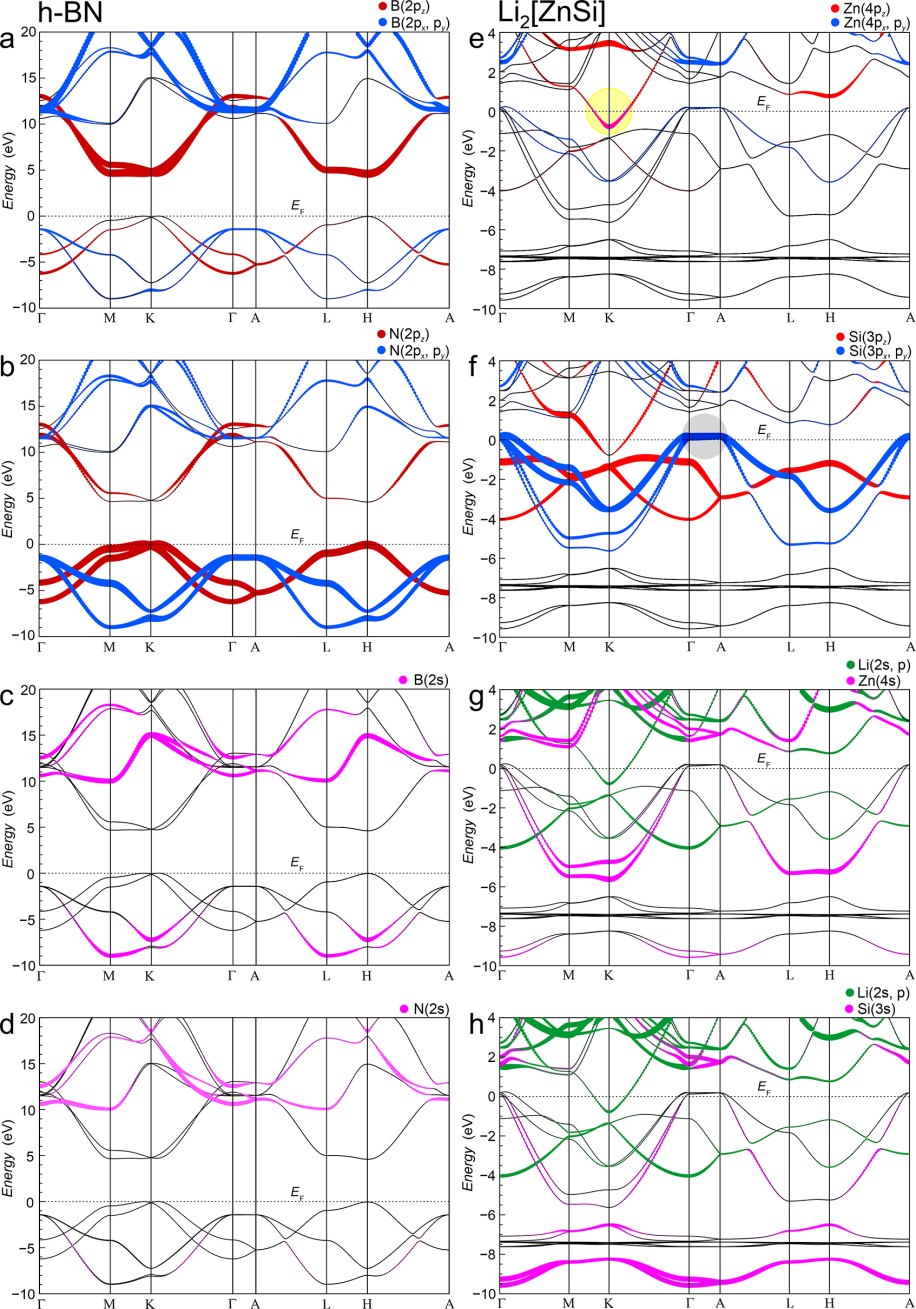
Comparison of fat-band structures of h-BN (a–d) and Li_2_[ZnSi] (e–h). Related species and their orbital projections are placed in the same rows. Li species in Li_2_[ZnSi] play an additional role and are depicted in diagrams (g) and (h). Li_2_[ZnSi] valence band maximum (VBmax) and conduction band minimum (CBmin) locations are emphasized by gray (f) and yellow (e) spheres, respectively.

The planar layers in both structures are located on crystallographic mirror planes such that the crystal orbitals can be strictly classified as σ- and π-type along most of the symmetry lines shown in Figure 3. The only exception is the line Γ-A, where this mirror symmetry of the little group of **k** at the end points is lost in-between.^21^ Nevertheless, the separation of σ- and π-type bands persists, even in this direction. The assignment employed leaves open whether the states are actually σ or π bonding, σ* or π* antibonding, or even nonbonding between nearest neighbors (1nn). With this simple distinction, the band structures of both compounds with 6 σ-type and 2 π-type valence bands along high-symmetry lines connecting high-symmetry **k** points of the hexagonal first Brillouin zone are similar enough such that their decisive differences can be well discussed.

A detailed analysis of the orbital-projected band structure (fat bands, Figure 3) reveals that the σ-bonding situation within the planar nets in Li_2_[ZnSi] is far from being optimal because the typical candidate states for strong in-plane σ-bonding, Si­(3p_*x*_, p_*y*_) and Zn­(4p_*x*_, p_*y*_), are energetically well separated, such that the unoccupied high-lying nominal Zn­(4p_*x*_, p_*y*_) states are only weakly mixing with one-half of the occupied Si­(3p_*x*_, p_*y*_) majority bands The overall rather asymmetric mixing Si–Zn is the band structure signature of their heteropolar bonding.

A more balanced mixing of the less-directed Zn­(4s) valence states (Figure 3g) with the Si­(3p_*x*_, p_*y*_) ones (Figure 3f) occurs at lower energies and at all **k** points shown except along Γ-A because of symmetry constraints. Along this direction, four unoccupied flat Si­(3p_*x*_, p_*y*_) derived uppermost “valence bands” are observed (gray circle, Figure 3f). They display only tiny Zn­(4p_*x*_, p_*y*_) and Li­(2p_*x*_, p_*y*_) stabilizing admixtures, while stabilizing in-plane Zn­(4s) contributions are prohibited by symmetry along this line^22^ (Figure 3g). They can be characterized as effectively nonbonding with respect to first nearest neighbors. Within the same plane, the Si atoms form six homoatomic second nearest neighbor contacts (*d*(Si–Si)^*xy*^ = |**a**| = 425 pm, Table 2), and the corresponding Si­(3p_*x,y*_)–Si­(3p_*x,y*_) in-plane overlaps along Γ-A are dominantly out of phase, i.e., antibonding. This feature is the reason for their rather high energies compared to the CBmin state with lower energy at K, with the result that they become depopulated.

In h-BN, this type of band along Γ-A can be identified as well. They are formed by the related N­(2p_*x*_, 2p_*y*_) types of states, but they are occupied in h-BN because their energy is lower than that of the uppermost π-type N­(2p_*z*_) valence bands (Figure 3b).

The CBmin state at point K in Li_2_[ZnSi] (yellow sphere in Figure 3e) corresponds to a Zn­(4p_*z*_) band that cannot mix with in-plane Si­(3p_*z*_) states for symmetry reasons.^22^ It is rather stabilized by σ-bonding interplane Zn­(4p_*z*_)–Zn­(4p_*z*_) interactions between second nearest neighbors (*d*(Zn–Zn)^*z*^ = 411 pm, Table 2) and by bonding nearest neighbor out-of-plane states of Li­(2p_*x*_, p_*y*_) (Figure 3f). This CBmin crystal orbital at K displays the complete intra- and intercell σ-bonding linear combination of Zn­(p_*z*_) orbitals; the corresponding antibonding combination is located at +4 eV. The matching 2-fold degenerate “nonbonding” crystal orbitals are found energetically in-between at about +1 eV at point H (Figure 3e), where both orbitals are degenerate due to intracell σ and intercell σ* p_*z*_–p_*z*_ bonding and vice versa. A band dispersion plot along K-H (not shown) would then show the approaching and final meeting of the two bands at H.

In h-BN, both types of stabilizing admixtures into the cationic p_*z*_ band at point K are not possible. The stabilizing role of the Li contributions is precluded by the absence of the separating layers. The homoatomic interaction along [001] is prevented by the *AA’* stacking of the layers. The heteroatomic interaction along [001] is secondary because of the large charge transfer gap between the occupied N­(2p) states and empty B­(2p) states. It can be related to the ionization energy difference given by −8.3 eV – (−13.2 eV) = 4.9 eV,^23^ which is quite well fulfilled in the DFT-PBE based band structure obtained.

In summary, it is the combination of high-lying second-nearest neighbor σ*-antibonding Si­(3p_*x*_, p_*y*_) orbitals along Γ-A in concert with the low-lying Li-stabilized σ-bonding Zn­(4p_*z*_) band at K that causes the negative VBmin-CBmax indirect band gap and, thus, metallic conductivity of Li_2_[ZnSi]. This valence-band depletion effect has some influence on Si–Zn bonding, as will be shown below.

#### Localization of Zn­(3d^10^) States

The “true” amount of Zn­(3d) participation in chemical bonding is difficult to assess as standard DFT functionals do not properly account for the localizing effects of electron correlation. It is known from elemental Zn that its hexagonal structure with a specific *c*/*a* lattice parameter ratio is difficult to reproduce using those functionals, which is attributed to the effects of the electron correlation not properly included.^24^ Moreover, the localizing correlation effect could be also dependent on the valency of Zn; i.e., the 3d^10^ states of ionic Zn^2+^ species could be more strongly localized than the ones for Zn^0^ species in elemental *hcp*-Zn and also for negatively charged Zn species in the present Li_2_[ZnSi] case (see below).

As a simple methodology to include the correlation of Zn­(3d) states, the PBE + *U* scheme was chosen. Several *U* values have been tested, namely, *U* = 0, 4, 6, 10, and 12 eV. It is found that with *U* = 0, the 3d^10^ states display a bandwidth of about 1 eV and are split into 2 DOS peaks (Figure 2 top) containing eight and two orbitals (Figure 4 top). The energetically higher lying two Zn­(3d) bands contain nominal σ-type 3d_*xy*_, 3d_*x*2–*y*2_, and 3d_*z*2_ states mixing with lower lying nominal Si­(3s) states. This way, the lower lying Si­(3s) and the higher lying in-plane Zn­(3d) states of σ-type symmetry are expected (perturbation theory) to display Si­(3s)–Zn­(3d) bonding and Zn­(3d)–Si­(3s) antibonding pairs, respectively. This feature is systematically suppressed upon increase of *U* and is barely noticeable at *U* ≥ 10 eV (Figure 4 bottom).

**Figure 4:**
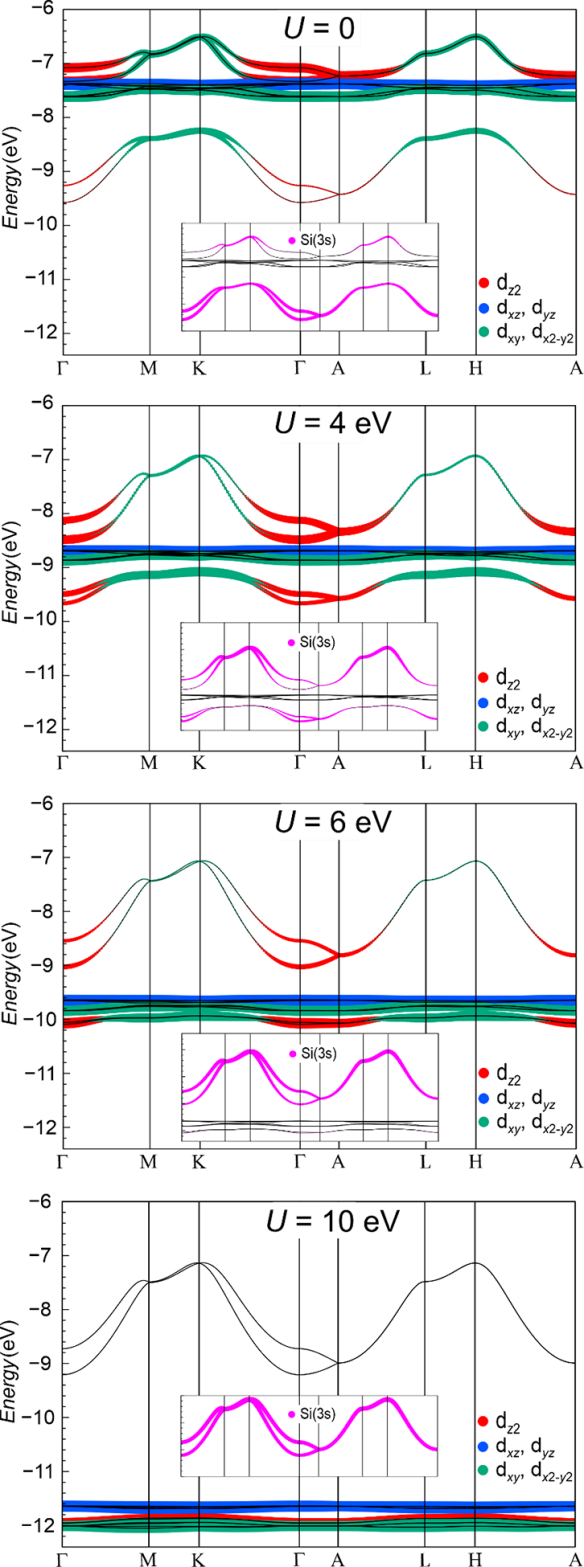
Increasing energetical localization of the Zn­(3d) bands (shown as fat bands) with increasing *U* values from 0 eV (top) to 4 and 6 eV (middle) and 10 eV (bottom). The location of the two nominal Si­(3s) bands switches from *E*[Si­(3s)] < *E*[Zn­(3d)] for *U* = 0 (top) to *E*[Si­(3s)] > *E*[Zn­(3d)] for *U* ≥ 4 eV (middle, bottom). They are identified from their larger dispersion combined with larger overall Si­(3s) contributions (see insets) and the systematic evolution with increasing *U*. The *U* dependence of the energetic sequence of the Si­(3s) and Zn­(3d) bands and their energy difference could be suitable for the experimental determination of a pertinent *U* parameter value with angle-resolved photoemission spectroscopy (ARPES).

#### Electronic Structure and Chemical Bonding in Position Space

The bonding situation in position space was analyzed within the electron-localizability approach^11^ using ELI-D and ED distributions computed from different wave functions obtained by variation of the PBE + *U* scheme (*U* = 0, 4, 6, 10, 12 eV). The QTAIM effective atomic charges (Li^+0.82^)_2_ [Zn^–0.17^ Si^–1.47^] obtained with *U* = 0 are in qualitative accordance with the Pauling electronegativity scale (Li (0.98), Zn (1.65), and Si (1.90)^25^) and the Allen one (Li (0.91), Zn (1.59), Si (1.92)^23^) but less clearly so with respect to the Allred–Rochow scale (Li (0.97), Zn (1.66), Si (1.74)^26^). The effect of the *U* value on these effective atomic charges turns out to be traceable but small in size (Table 3). The observed decrease with increasing *U* is opposite to the anticipated effect caused by increasing localization of the Zn­(3d) states. Instead, the decreased charge transfer is assigned to the volume reduction of the Zn atoms (Table 3). Obeying the sum rule of QTAIM atomic volumes to reconstruct the unit-cell volume, the volume shrinkage of Zn atoms is found to be mostly compensated for by the Si atoms, while the Li atoms remain rather unaffected.

**Table 3:** Effect of *U* Values (in eV) on Position Space Bonding Features Obtained by Integration of the Electron Density^a^

	*U* = 0	*U* = 4	*U* = 6	*U* = 10	*U* = 12
*Q* ^eff^(Li), *V*(Li)	+0.82, 4.10	+0.81, 4.12	+0.81, 4.13	+0.81, 4.14	+0.81, 4.15
*Q* ^eff^(Zn), *V*(Zn)	–0.17, 21.81	–0.13, 21.54	–0.12, 21.37	–0.10, 21.03	–0.09, 20.88
*Q* ^eff^(Si), *V*(Si)	–1.47, 34.22	–1.49, 34.46	–1.50, 34.62	–1.52, 34.93	–1.53, 35.06
*N* ^ELI‑D^(bond), *V* ^ELI‑D^(bond)	4.21, 28.87	4.17, 27.82	4.15, 27.78	4.11, 27.69	4.09, 27.65

a First item of each box: the QTAIM effective atomic charge *Q* ^eff^(*A*) or the electronic ELI-D basin population *N* ^ELI‑D^(bond); the second item of each box gives the associated volume (in 10^6^ pm^3^).

##### Topological Coordination Numbers

A detailed evaluation of the coordination situations of all three species is obtained from the shapes of the topological atoms in position space calculated from the electron density distribution (QTAIM methodology). In the topological coordination number (tCN) approach,^18^ the solid angles subtended by each diatomic contact surface (interatomic surface, IA surface) at the corresponding nuclei are employed. The requirement of fulfilling the condition of coordination reciprocity leads to a sequence of sub-coordination scenarios denoted as *scene* with increasing overall coordination, which are characterized by a specific overall weight ^sc2^ *W*_*scene*_^°^ and effective coordination numbers of the species. The weights are related to the separation with respect to the subsequent coordination scenario with overall higher coordination, and the associated effective coordination numbers of each species are related to the sum of decreasing coordination increments from the neighbors included.

As a result of the procedure (Table 4, Figure 5), the sub-coordination scenario with the 3-connected _∞_^2^[ZnSi] partial structure with distance *d*(Si–Zn)^*xy*^ = 245 pm (Tables 2 and 4) is found with the overall highest weight of 1 (scenario “*max*”). A similar result is obtained for h-BN (Table 5). For Li_2_[ZnSi], it is closely followed in importance by scenario “*max–1*” with only slightly lower weight of 0.94, where additionally the Si–Li^*z*^ contacts with distances *d*(Si–Li)^*z*^ = 274 pm (Tables 2 and 4) are included. The scenario “*max–2*” with the third highest weight of 0.81 includes contributions of slightly longer Si–Li^*xyz*^ distances *d*(Si–Li)^*xyz*^ = 281 pm (Tables 2 and 4). The resulting 11-coordination of Si corresponds to the Edshammar polyhedron discussed for alkaline metal pnictides *A*_3_ *Pn* adopting Na_3_As-type crystal structures.^27^ Its characterizing apical vs lateral extension ratio amounts to *c* ^Eds^/*a* ^Eds^ = 1.29. This value is higher than the ∼1.18 reported for various *A*_3_ *Pn* (alkali-metal pnictides) compounds.^27^

**Figure 5:**
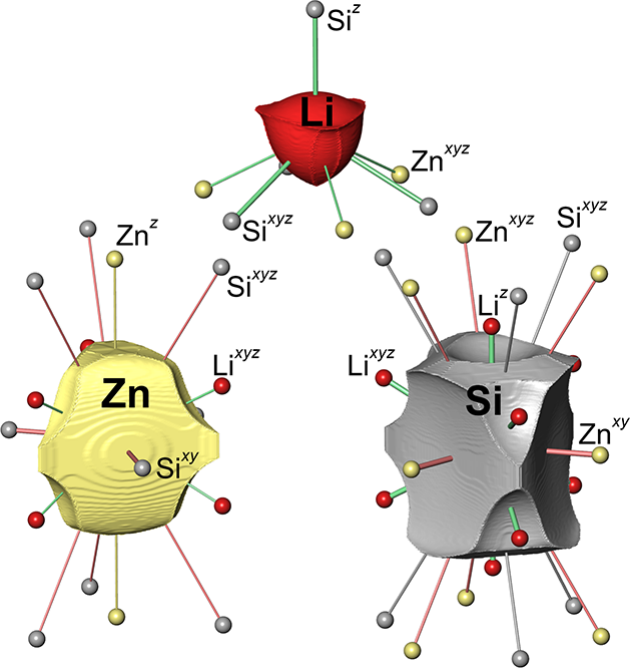
QTAIM atomic basins and topological coordination displaying all neighbors according to scenario “*tot*” (Table 4). The neighbors included in scenario “*max–2*” are indicated by thicker connection lines.

**Table 4:** Li_2_[ZnSi] Topological Coordination Scenarios^a^

s*cene*	Li coord., *tCN*_*scene*_(Li), ${tCN}_{\mathrm{scene}}^{\mathrm{eff}}\left(\mathrm{Li}\right)$	Zn coord., *tCN*_*scene*_(Zn), ${tCN}_{\mathrm{scene}}^{\mathrm{eff}}\left(\mathrm{Zn}\right)$	Si coord., *tCN*_*scene*_(Si), ${tCN}_{\mathrm{scene}}^{\mathrm{eff}}\left(\mathrm{Si}\right)$	^sc2^ *W*_*scene*_^°^
*max*	[./.], 0, 0	[0Li, 3Si], 3, 3.00	[0Li, 3Zn], 3, 3.00	1
max–1	[0Zn, 1Si], 1, 0.72	[0Li, 3Si], 3, 3.00	[2Li, 3Zn], 5, 4.44	0.94
max–2	[0Zn, 4Si], 4, 2.01	[0Li, 3Si], 3, 3.00	[8Li, 3Zn], 11, 7.03	0.81
max–3	[3Zn, 4Si], 7, 2.51	[6Li, 3Si], 9, 3.99	[8Li, 3Zn], 11, 7.03	0.22
*tot*	[3Zn, 4Si], 7, 2.51	[6Li, 9Si; 2Zn], 17, 4.24	[8Li, 9Zn; 6Si], 23, 7.67	0.02

a The most important coordination scenarios denoted as *scene* are listed with increasing coordination numbers; scene notations *max*, *max–1*, *max–2*, and *tot* indicate scenarios with the highest, second highest, and third highest normalized scenario weights ^sc2^ *W*°_*scene*_ and the total coordination scenario *tot* (Figure 5), respectively. In each box, the coordinating neighbors of each species in the specific scene (first item), the resulting coordination number *tCN*_*scene*_ (second item), and effective coordination number ${tCN}_{\mathrm{scene}}^{\mathrm{eff}}$ (third item) are given.

**Table 5:** Topological Coordination Numbers for h-BN^a^

*scene*	B coord., *tCN* *_scene_* (B), *tCN*_*scene*_^eff^(B)	N coord., *tCN* *_scene_* (N), ${tCN}_{\mathrm{scene}}^{\mathrm{eff}}\left(N\right)$	^sc2^ *W*_*scene*_^°^
*max*	[3N], 3, 3.0	[3B], 3, 3.0	1
max–1	[3N], 3, 3.0	[3B; 6N], 9, 3.60	0.12
max–2	[3N], 3, 3.0	[3B; 12N], 15, 4.03	0.08
*tot*	[5N], 5, 3.12	[5B;12N], 17, 4.15	0.01

a The three heteroatomic contacts within the 6^3^ nets (*d*(N–B)^*xy*^ = 145 pm) correspond to the scenario (”*max*”) with maximal weight ^sc2^ *W*_scene_^°^. Additional homoatomic N–N coordination between the layers (*d*(N–N)^*xyz*^ = 363 pm, “*max–1*”) and within the layers (*d*(N–N)^*xy*^ = 250 pm, “*max–2”*) are found to display a higher weight than the total coordination scenario (”*tot*”) with additional heteroatomic coordination between the layers (*d*(N–B)^*z*^ = 333 pm).

The subsequent coordination scenario with higher overall coordination displays a clearly lower weight of 0.22 (”*max–3*”, Table 4). We note here that the six Zn–Li^*xyz*^ contacts of *d*(Zn–Li)^*xyz*^ = 281 pm display a lower effective coordination increase of Δ*tCN* ^eff^(Zn) = 3.99–3.0 = 0.99 than the six equidistant Si–Li^*xyz*^ contacts with Δ*tCN* ^eff^(Si) = 7.03–4.44 = 2.59 (Table 4). In this case, the different sizes of the corresponding IA surfaces, i.e., IA­(Zn–Li) < IA­(Si–Li) (Figure 5), also lead to a similar solid angle gradation that is directly associated with the effective coordination increments of both contact types observed.

The scenario “*tot*” containing all interatomic contacts is found to display the lowest weight because of the small solid angles of the last contacts included. For a visual understanding of the most important scenarios, it is advantageous to inspect the interatomic surfaces (Figure 5). The most important contact (scenario “*max*”) is the Zn–Si one displaying a large convex IA surface at the Zn atom and a correspondingly concave one at the Si atom. Typically, the concave IA surfaces are found at the anionic side of the cation–anion contact. In the present case, both Zn and Si species are negatively charged, but the Zn atom is significantly less so, and it displays a smaller volume (Table 3).

The Li cations, being the smallest and least polarizable species, display solely convex IA surfaces. A view on the Li atomic shape finds a roughly hexagonal pyramidal shape in agreement with its overall (scenario “*tot*”) 7-fold coordination.

The _∞_^2^[Zn^0.17–^Si^1.47–^]^1.64–^ layers are stacked in the *AB* sequence and are separated from each other by a layer of Li^0.82+^ cations (Figure 1a), which implements favorable ionic interactions along [001].

The effective electron transfer from Li to the layer atoms is found to be different from the conceptual situation [Zn^2–^ Si^0^]^2–^, which would be ideally related to a graphene sheet. The observed effective charge distribution points to an electronic situation more related to that of h-BN, i.e., [B^*x*+^ N^*x*–^]. It leads to a severe underpopulation of the Zn­(4p_*x*,__*y*_) atomic states that would be required for realizing a saturated sp^2^ type of σ-bonded covalent network. On the other hand, Si^1.47–^ species have 1.47 electrons too many for a graphene type of delocalized π-bonding. This excess is not counterbalanced by the corresponding number of holes according to Zn^(2–1.47)–^ because the lithium species Li^0.82+^ has not transferred its valence electron completely. We like to note here that tetrel elements *Tt* with an even smaller electronic population, e.g., Si^1.28–^ in CaSi^28^ and Ge^1.26–^ in CaGe,^29^ form 2-bonded tetrel partial structures (2b)*Tt* ^2–^ holding two lone pairs. They were actually found to form polar-covalent *Tt*–*cation* bonding via the lone pairs as explained within the polarity-extended 8–*N* ^eff^ rule framework.^29,30^ For Li_2_[ZnSi], this means that the Si π-type 3p_*z*_ atomic states are overpopulated with respect to the situation in graphene and similar to N^*x*–^ anions in BN. Moreover, they are not involved in covalent interactions with the in-plane Zn atoms with low electronic populations and virtually unavailable Zn­(4p_*z*_) atomic states (except the CBmin state at K), as shown in the band structure analysis. Thus, it is consistent that specifically, the Si^1.47–^ anions display the higher 2 + 6 coordination by Li^0.82+^ cations (scenario “*max–2*”, Table 4) compared to Zn^0.17–^ being only 6-coordinated by Li^0.82+^ at lower weight and with lower effective coordination number *tCN*_max–3_^eff^(Zn) = 3.99 vs *tCN*_max–3_^eff^(Si) = 7.03 (scenario “*max–3*”, Table 4). The corresponding primarily heteroionic coordination solution in h-BN is achieved by the *AA*’ stacking of the [BN] layers as explained before.

##### Covalent Bonding from ELI-D Analysis

For characterizing the covalent bonding situation, topological analysis of the ELI-D distribution Υ_*D*_^σ^(**r**) in position space^11^ was employed. As a result, only one type of local ELI-D maximum is observed in the valence region of position space (Figure 6 top). These maxima are located above and below the Si atoms along the vertical line Si–Li^*z*^ being symmetrically related to each other via the horizontal mirror plane in which Si and Zn species are located. The electron count for each of the associated ELI-D basin regions amounts to 4.2 e^–^ (Table 3). This is interpreted as undistinguishably merged π-type lone pair and σ-bonding electronic regions, resulting in the maximal electron localizability located above and below the [ZnSi] planes. For comparison, h-BN displays local ELI-D maxima along the three N–B contacts (Figure 7 top) and above and below each N atom. However, only the polar-covalent N–B contacts feature a two-atomic ELI-D basin with electronic population of 2.59 e^–^, while the tiny basin regions above and below the N atoms contain only 0.007 e^–^, which is an amount beyond chemical significance. Their presence indicates a weak tendency for creating a lone pair type of ELI-D feature in h-BN as well. Thus, the lack of a well-defined Si–Zn ELI-D 2-atomic bonding basin reveals strongly different intralayer bonding situations in Li_2_[ZnSi] and h-BN. This may be caused either by the antibonding effect of the filled Zn­(3d^10^) orbitals in the valence region or by band structure effects leading to the depopulation of 4 σ-type Si­(3p_*x*_, p_*y*_) orbitals along Γ-A as discussed above.

**Figure 6:**
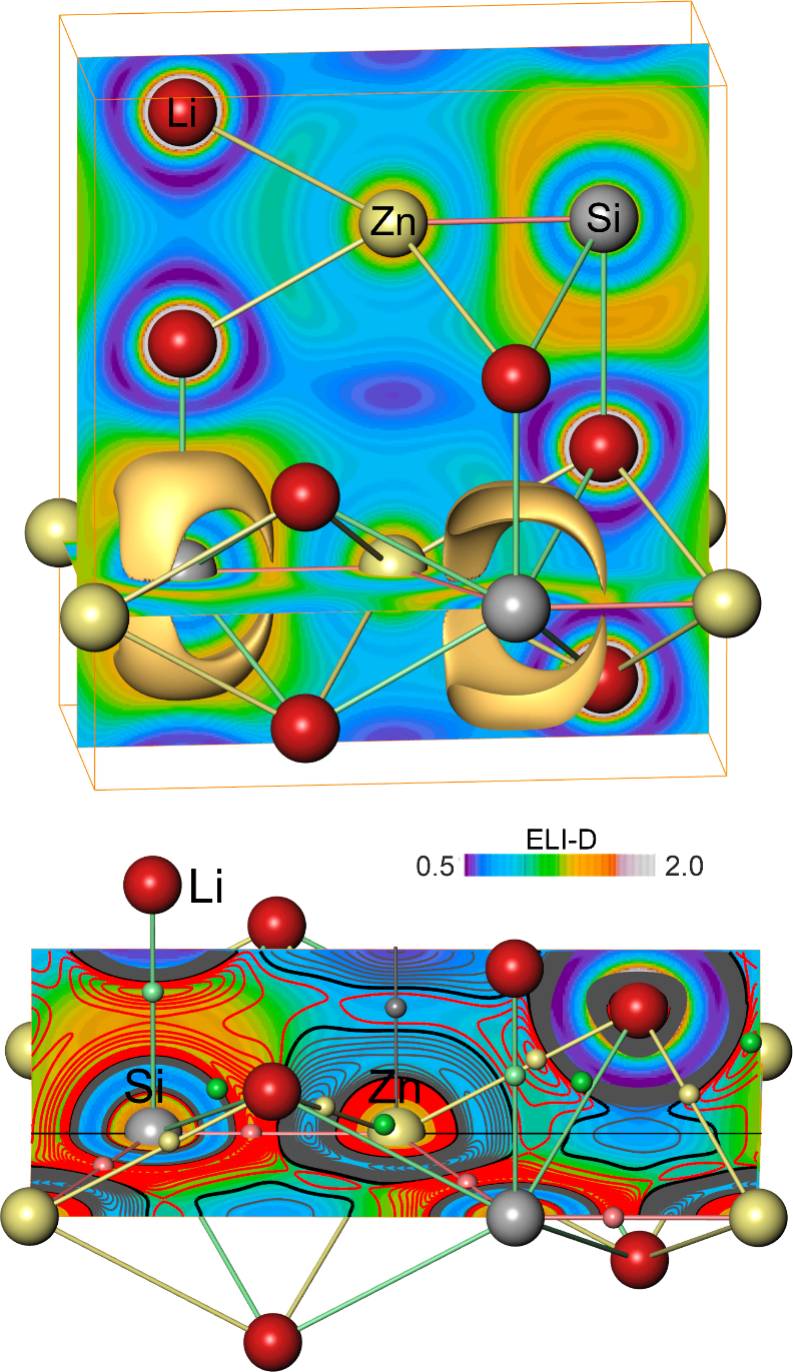
(Top) ELI-D diagram displaying two slice planes (see the colormap) and four isosurfaces (ELI-D value 1.37) and (bottom) negative relative ELI-D Laplacian distribution −∇^2^Υ_D_^σ^(**r**)/Υ_D_^σ^(**r**) shown as isolines drawn on the ELI-map; positive isoline values are drawn in red; negative ones in blue color; the 0-value isoline is drawn in black. Local maxima are indicated by small spheres in the color of the contact lines assigned. The ELI-D colormap is valid for both diagrams.

**Figure 7:**
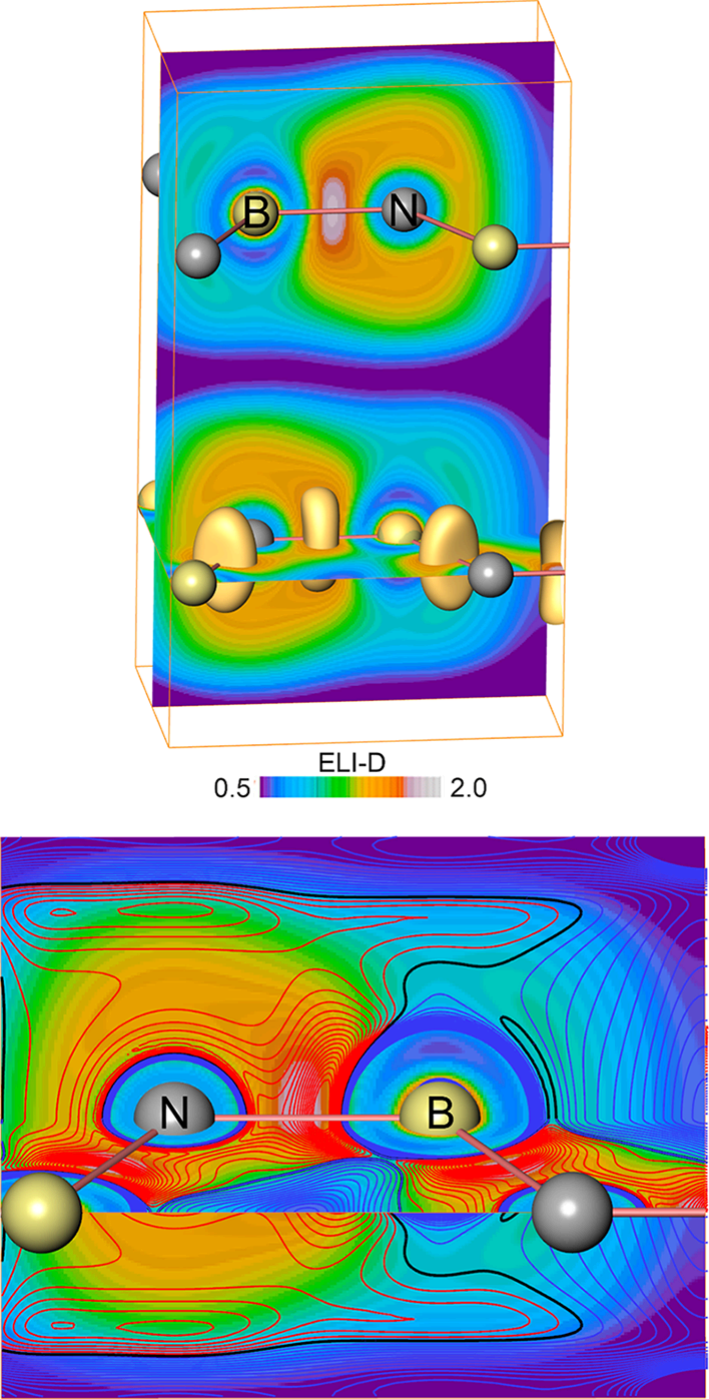
(Top) ELI-D diagram for h-BN. ELI-D values (see colormap) and isosurfaces at value 1.55 are shown to display N–B bond basin locations. ELI-D local maxima above and below N atoms would be present at an isosurface value of 1.4386 but would be too small to visualize. (Bottom) Positive and negative values of the negative relative ELI-D Laplacian −∇^2^Υ_D_^σ^(**r**)/Υ_D_^σ^(**r**) are shown by red and blue isolines, respectively, on top of the ELI-D map.

It is instructive to note that with an overall decrease of 0.12 e^–^ (3%), the populations of the ELI-D valence basins in Li_2_[ZnSi] are only slightly affected by *U* (Table 3), while the topology remains unaffected. Thus, the reason for the difference is not the participation of the d orbitals but rather the holes in the valence band.

In Li_2_[ZnSi] with Si being surrounded by two valence basins, a value of 4 e^–^ per valence basin would be the formally correct value for fulfillment of the octet rule in position space.^30^ The slight deviation of 0.21 e^–^ from 4 (Table 3) is related to the deviation of the position-space core–shell populations of each atom obtained from ELI-D space partitioning from the integral orbital shell populations in Hilbert space.^31^ The decreasing deviation with increasing *U* indicates that the valence region overpopulation in Li_2_[ZnSi] is caused to a larger extent by the nonlocalized Zn­(3d^10^) states for *U* = 0. With the absence of ELI-D maxima between the in-plane nearest neighbor contacts of Si–Zn, the ELI-D topology does not display the primary indicator for covalent Si–Zn bonding. A similar observation has been previously reported for other Si–Zn bonding situations in the framework of ELF (electron localization function^32^) analysis.^33^ In the present case, a more detailed topological analysis yields an ELI-D (3, +1) saddle point along each internuclear Si–Zn line, which is flanked by two (3, −1) saddle points located in the perpendicular direction within the plane. The (3, −1) points represent the basin interconnection points (*bip*s^34^), where the two valence basins above and below the plane touch with the highest ELI-D value. All three ELI-D critical points are located within the QTAIM region of the Si atoms as expected, since it represents the species with the effectively highest electronegativity, as indicated by the QTAIM effective charge. The reduced mixing of in-plane Si and Zn valence states detected from the fat-band structures (Figure 3e–g) leads to a special polar-covalent type of bonding situation that is not indicated by an ELI-D local maximum between Si–Zn.

For these types of cases, analysis of the fine structure of the ELI-D distribution has been proposed to distinguish covalently weaker bonded from nonbonded atomic contacts.^35^ For this purpose, the distribution of the relative Laplacian of ELI-D −∇^2^Υ_*D*_^σ^(**r**)/Υ_*D*_^σ^(**r**) is computed, and the regions of positive values are analyzed. These are the regions that display a similar but more general signature for a covalently bonded situation between atoms than the ELI-D maxima primarily do, namely, negative relative ELI-D Laplacian values. Note that the ELI-D Laplacian measures the average curvature of ELI-D at each point; at a local maximum, the curvatures are negative in all three directions, while a negative average curvature indicates an overall dominance of the negative curvatures over the positive ones. In the framework of charge density ρ­(**r**) analysis, the quantity −∇^2^ρ­(**r**) is called charge concentration^10^ following Morse and Feshbach.^36^ This mathematical interpretation of the Laplacian of a scalar field like Υ_*D*_^σ^(**r**) does not change upon renormalization of the average curvature of ELI-D by the positive ELI-D value. Recently, it has been found that directed weaker covalent bonding situations can be detected from the local maxima of the negative relative ELI-D Laplacian −∇^2^Υ_D_^σ^(**r**)/Υ_D_^σ^(**r**) along the bond line.^29^

In Li_2_[ZnSi], there is a fully connected region of negative relative ELI-D Laplacian values in the valence region between all species (Figure 6 bottom). The related diagram for h-BN is given in Figure 7 bottom. Specific for pairwise bonding, the local maxima of this quantity are found (Figure 6 bottom) between in-plane Si–Zn^*xy*^ (value 3.01), out-of-plane Si–Li^*z*^ (value 1.66), Si–Li^*xyz*^ (1.10), Zn–Li^*xyz*^ (1.15) contacts, and a double maximum along Zn–Zn^*z*^ (value 0.41). The behavior of all these maxima with increasing *U* is analyzed in Appendix A. The most important result with respect to the covalent bonding in the [ZnSi] partial structure is that the Zn–Si contact displays the highest −∇^2^Υ_D_^σ^(**r**)/Υ_D_^σ^(**r**) value, and it is the only one that clearly increases with *U* (Figure 8) indicating that the nonlocalized Zn­(3d^10^) states have a negative influence on Zn–Si ELI-D attractor formation along the contact line. However, an ELI-D maximum for the diatomic Zn–Si contact is not obtained even at *U* = 12 eV. Therefore, the decisive effect impeding this attractor is not the nonlocalized Zn­(3d^10^) states, but the Si­(3p_*x*_, p_*y*_) valence-band depletion along Γ-A (Figure 3f). In contrast, related σ-type N­(2p_*x*_, p_*y*_) band states are fully occupied in h-BN in the complete Brillouin zone (Figure 3b).

**Figure 8:**
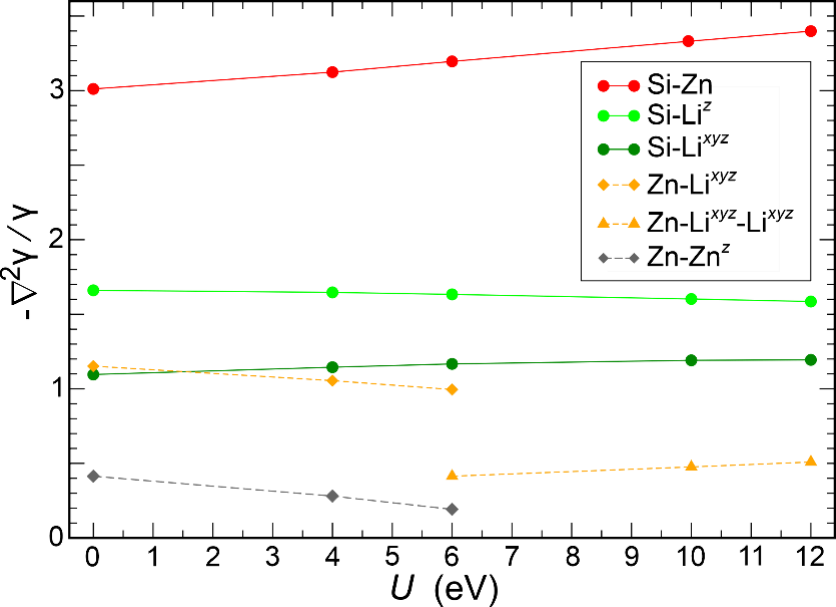
Variation of −∇^2^Υ_*D*_^σ^(**r**)/Υ_*D*_^σ^(**r**) values at different local maxima as a function of *U* values (0, 4, 6, 10, and 12 eV) applied.

Even with only one type of ELI-D valence basin, the atomicity of the bond basins, i.e., the number of atomic basins contributing a certain amount to the electronic population of the ELI-D basin, can be determined via the ELI-D/QTAIM basin intersection method.^11^ Each of these valence basins is intersected by 8 (1Si, 3Zn, 4Li) QTAIM atomic basins, which means that it is 8-atomic, denoted as 8a-(1Si, 3Zn, 4Li). Noteworthy, each of these contacts is also marked by a negative relative Laplacian local maximum as discussed before. With 2.69 e^–^ (64%), the Si atom is the majority contributor to the bond basin population. The three 1nn Zn neighbors each contribute 0.46 e^–^ (11%). The four Li neighbors contribute only very small amounts with 0.043 e^–^ from vertical Li and 3 × 0.029 e^–^ from three further Li neighbors, which sums to 3% of the total population. Such small contributions are typically neglected in a conceptual view of the bonding situation. The valence basins are then considered as effectively four-atomic 4a^eff^-(1Si, 3Zn) polar-covalent Si–3Zn bonds. In view of the indications from the local maxima of the relative ELI-D Laplacian, the small contributions are mentioned here as well, in order to demonstrate consistency of the picture. They occur at the lowest diatomic attractor values of the negative relative ELI-D Laplacian (Figure 8), and they are found to contribute the lowest number of electrons in the ELI-D/QTAIM basin intersection. This behavior consistently ranks their role in covalent bonding with other species as the second weakest bonding effect among the ones discussed. The weakest one is the occurrence of the negative Zn–Zn^*z*^ ELI-D Laplacian attractor for *U* ≤ 6 eV.

## Conclusions

Li_2_[ZnSi] is an intermetallic compound with a [ZnSi] partial structure that fits into the Zintl scheme according to a bonding situation formally related to (Li^+^)_2_[ZnSi]^2–^ featuring a 2D polyanionic partial structure isoelectronic to graphene. This is different from structurally related *A*_3_ *Pn* compounds, which are formally written as (*A* ^+^)_3_ [*Pn* ^3–^] symbolizing isolated pnictide anions coordinated by alkali metal cations. Real-space analysis of the bonding situation in Li_2_[ZnSi] reveals that Zn is negatively charged, which corroborates the 2D partial structure scenario related to that of graphene and its polar-covalent variant h-BN. Nevertheless, comparative bonding analysis of Li_2_[ZnSi] and h-BN reveals also a clear difference. The major point is not the incomplete charge transfer from Li to the [ZnSi] partial structure, which is a feature observed for many compounds fitting into the Zintl–Klemm scheme.^28,29,37^ It is the stabilizing influence of the Li^0.82+^ cations on the π-type valence and conduction bands and homoatomic interlayer Zn–Zn^*z*^ interactions that lead to a disruption of the typical semiconductor band structure (like that of h-BN) by forming a negative indirect band gap and metallic conductivity via population of Li­(2p)-stabilized Zn­(4p_*z*_) π-type conduction band states (at K) and concomitant depopulation of σ-type Si­(3p_*x*_, p_*y*_) valence bands (along Γ-A). This easily identifiable band structure effect leads to a vanishing of the diatomic N–B type of real-space ELI-D bond maximum Si–Zn in Li_2_[ZnSi] such that the polar covalent Zn–Si 4-atomic bonding obtained can be only analyzed in more detail by using the local maxima of the negative relative ELI-D Laplacian as a secondary bonding indicator in the ELI-D framework.

The decision whether Li_2_[ZnSi] can be classified as a Zintl phase is clearly beyond the scope of quantum chemical analysis. A phase qualifies as a Zintl phase if its crystal structure can be explained by formal electron transfer from the least electronegative atoms in the compound to the more electronegative ones and subsequent application of valence rules relating the resulting formal electron counts to the partial structures actually identified.^38^ Within this conceptual framework, Li_2_[ZnSi] is classified as a Zintl phase. Its unusual bonding situation within the polyanion that is related to the electronic situation at the Fermi level represents an example of a type of bonding scenario that is still in line with the basic Zintl concept.

## Appendix A

### Effect of Zn­(3d^10^) Correlations on the Relative ELI-D Laplacian Maxima and ELI-D Core–Shell Structuring

The local maxima of −∇^2^Υ_*D*_^σ^(**r**)/Υ_*D*_^σ^(**r**) in Li_2_[ZnSi] (values in Figure 8) for *U* = 0 are found between in-plane Si–Zn^*xy*^ (value 3.01), out-of-plane Si–Li^*z*^ (value 1.66), Si–Li^*xyz*^ (1.10), Zn–Li^*xyz*^ (1.15) contacts (Figure 6 bottom), and a double maximum along Zn–Zn^*z*^ (value 0.41). Besides two local relative Laplacian maxima along Zn–Zn^*z*^ in the [001] direction, there is no other such feature between the homoatomic contacts in this compound. The double maximum along Zn–Zn^*z*^ with a comparably low negative relative Laplacian value (0.41) is considered as the weakest type of secondary bonding indicator, because these maxima are separated by a region of positive relative Laplacian values (Figure 6 bottom), which hints to interpret them as a trace of the fully occupied Zn­(3d_*z2*_) and partially occupied Zn­(4p_*z*_) states being indicated by some kind of partial lone pair character. It has vanished for *U* > 6 eV (Figure 8). Nevertheless, together with the Zn–Li^*xyz*^ negative relative ELI-D Laplacian maxima, they may be considered as weak real-space indications of the decisive σ-type Zn­(4p_*z*_)–Zn­(4p_*z*_) and Zn­(4p_*z*_)–Li­(p_*x*_, p_*y*_) orbital interactions in the [001] direction, leading to the characteristic CBmin state at point K of the 1st Brillouin zone.

The increase of the Zn­(3d) *U* value along 0, 4, 6, 10, and 12 eV leads to a systematical decrease of the relative ELI-D Laplacian value along Zn–Li^*xyz*^ (Figure 8), and for *U* = 6 eV, an additional maximum appears within the triangle Li–Zn–Li. For *U* ≥ 10 eV, only this maximum remains at a low value of about 0.5. Likewise, the Zn–Zn^*z*^ double maximum is also affected by *U* as already mentioned before.

The Zn-related ELI-D Laplacian features in the valence region just discussed also have a signature directly at the Zn atoms themselves, namely, the amount of Zn penultimate shell structuring measured by the structuring index *ε*.^34^ It is expected to decrease toward 0 on increasing the value of *U*. This would indicate the absence of genuine 3rd shell orbital contributions like the Zn­(3d) ones in covalent bonding features in the valence-shell region. It is found that the 3rd shell structuring even without *U* is quite small (ε = 0.011), displaying already a certain intrinsic degree of localization provided by the DFT-PBE functional employed. The increase of Zn­(3d) *U* values along *U* = 0, 4, 6, and 10 eV yields the expected effect of decreasing Zn 3rd shell structuring-index values ε = 0.011, 0.010, 0.009, and 0.007. At *U* = 12 eV, the precision of ε determination on the grid is too small, such that the effect is at the boundary of investigation on the 1.67 pm grid mesh-size employed. In summary, the decrease of the Zn 3rd shell structuring index *ε* in position space represents a position-space counterpart to the Zn­(3d^10^) orbital localization picture given in Figure 4.
